# Toward a better understanding of phylogenetic relationships within Conringieae (Brassicaceae)

**DOI:** 10.22099/MBRC.2022.42767.1709

**Published:** 2022-03

**Authors:** Ahmad Reza Khosravi, Atena Eslami-Farouji, Atiqullah Sultani-Ahmadzai, Sasan Mohsenzadeh

**Affiliations:** 1 Department of Biology, School of Science, Shiraz University, Shiraz, Iran; 2 Department of Biology, Paktia University, Faculty of Science, Afghanistan

**Keywords:** Arabis ottonis-schulzii, Brassicaeae, Conringia, Plagioloba, Plagiolobeae

## Abstract

One new tribe (Plagiolobeae), one new species (*Plagioloba derakii*) together with two new combinations (*P. persica and P. clavata*) are established within Brassicaceae based on a decisive consideration of molecular phylogenetic dataset, morphological characters, fruit septum nature, as well as seed microsculpturing features. Results distinctly justified *Arabis ottonis-schulzii* as a synonym of *Conringia persica* and further molecular analyses proved its placement as a member of genus *Plagioloba*. It is also placed in a new tribe Plagiolobeae as close relatives of Conringieae and Coluteocarpeae. Finally, the diagnostic morphological characters separating the new tribe from the previously assigned tribe (Conringieae) are also discussed.

## INTRODUCTION

Generic delimitations of some genera within Brassicaceae have still been representing critical confusion [e.g., 1]. Detailed studies have been revealed high levels of paraphyly or polyphyly in Brassicaceae [e.g., 2-7]. As the relationships of unresolved taxa have not yet been botanically and phylogenetically explored, every single enigmatic genus within the family Brassicaceae (e.g., *Arabis *L.) might demonstrate different independent lineages with artificial boundaries [2]. Consequently, it is possible to treat strongly supported clades as a separated taxon [8 and references therein].* Arabis* is one of the problematic genera with about 60 to 180 species worldwide [e.g., 2, 9-11 and references therein] due to variation in morphological characters [12]. It is first studied historically by Hopkins and continuously examined by different authorities [9 and references therein]. Schulz generated an artificial sectional classification, which later subsumed under *Arabidopsis *[13]. Although different studies considered *Arabis* species in their phylogenetic framework [e.g., 3, 6, 14], none of which disclose the distinct taxonomic position of remained unresolved taxa. Koch and Grosser believed that the systematic position of only a few *Arabis* species remained vague, and such kind of enigmatic taxa can be assigned in different genera and tribes [12]. Nikolov et al. tried to understand the evolutionary position of an overlooked taxon, *Arabis ottonis-schulzii *Bornm. & Gauba, but their data were not sufficient to determine its true taxonomic position [15]. *Arabis* *ottonis-schulzii* was described by Bornmüller and Gauba based on two specimens collected by Gauba in the Kalak region and Kuh-e Dashteh near Karaj city in Iran [16]. It is an annual endemic Iranian plant, which is mainly distributed in Iran and Afghanistan mountains.

Our story just began when the first author of this paper (ARKH) paid attention to the morphological similarities between *Arabis* *ottonis-schulzii* and *Conringia *Heist. ex Fabr,. On the other hand, he noticed that both species were subsumed under *Conringia persica *Boiss. in Flora of Iran in Persian [17]. Consequently, he carefully checked the Flora of Iran and plants in the field and distinguished that *C. persica* shows two different variants. One is similar to the type specimens which were collected by Kotschy in Shiraz, Kuh-e Barfi, and the second one shows different morphological characters, which will be discussed later. 

According to German and Al-Shehbaz, *Conringia* is mainly centered in Iran and Turkey with ca. 6 species [18]. The tribal assignment of *Conringia* was revised by various taxonomists [e.g., 13, 19- 27]. *Conringia* was initially placed in tribe Sisymbrieae by Bentham and Hooker [19], while Prantl [20] arranged *Conringia* species in tribe Hesperideae and subtribe Moricandiinae. Schulz, Hayek, Janchen and Kamelin assigned *Conringia* in tribe Brassiceae and subtribe Moricandiinae [13, 21, 22, 25]. Gómez-Campo initially in [28] and later in [24], assigned *Conringia* in Brassiceae and in his latter study considered *Conringia* and *Calepina* Adans. as closely related genera. Earlier, Botschantzev temporarily disagree with the placement of *Conringia* in Brassiceae, but he did not find enough clues to put it in different tribe [23], while Al-Shehbaz questioned the validity of *C. persica* within Brassiceae due to the absence of conduplicate cotyledons and beaked fruits [29]. Warwick and Sauder preserved *Conringia* within Brassiceae [30], but later workers did not follow this idea [e.g., 31-34]. Earlier, the first author of this paper (ARKH) unraveled the true taxonomic position of *Iljinskaea planisiliqua* (Fisch. & C.A. Mey.) Al-Shehbaz, Özüdoğru & D.A. German (previously known as *Conringia planisiliqua* Fisch. & C.A. Mey.) as the sister group for Isatideae in his Ph.D. thesis [35]. Beilstein et al. confirmed the displacement of *Conringia persica* to the tribe Brassiceae, and supported the assignment of it to Coluteocarpeae with high clade credibility [33]. Al-Shehbaz et al. plus subsequent treatments (see [27] and references therein) were not followed the previous tribal assignment for *Conringia* [26] and left it unresolved [e.g., 34, 36, 37]. Finally, German and Al-Shehbaz together with German et al. provided enough evidence to place *Conringia* together with *Zuvanda* (Dvořák) Askerova in tribe Conringieae [18, 27]. Simultaneously, Khosravi et al. indicated the distinct position of *I. planisiliua* within tribe Conringieae [38]. However, he formerly recognized the true taxonomic position of *I. planisiliua* and *C. orientalis* in his Ph.D. thesis [35]. His idea was later confirmed by Liu and his colleagues, as they proved the close relationship of *C. planisiliua* with the tribe Isatideae [5]. Nevertheless, they misleadingly suggested the placement of *Conringia* within tribe Isatideae. Some years later, Al-Shehbaz et al. clarified *C. planisiliua* true taxonomic position and introduced a new genus named *Iljinskaea *Al-Shehbaz, Özüdoğru & D.A. German (Isatideae) [39]. 

*Zuvanda* is mainly distributed in Southwest Asia with three species [18]. In the beginning, *Zuvanda* was placed within *Malcolmia* by Schulz [13], but this idea was in contrast with Dvořák statement [40]. He transferred *Zuvanda* to a different genus (*Maresia* Pomel.). Askerova declared that *Zuvanda* morphologically differs from *Malcolmia* by the absence of trichome (or simple small hairs), presence of auriculate, and amplexicaul stem leaves [41]. On the other hand, Dorofeyev believed that *Zuvanda* and *Conringia* *clavata* Boiss. were belonging to *Moricandia* DC. [42], but this idea did not receive the attention of the followers [26, 30, 31, 43] due to unlike cotyledons. Warwick et al. tried their best to show *Zuvanda* relationship with *Goldbachia* *laevigata* (M. Bieb.) DC. due to annual life form, lack of hair (glabrous), leaf shape, and glaucous color [44]. They clearly proved that *Zuvanda* is nothing to do with tribes Anchonieae, Chorisporeae, Euclidieae and Hesperideae, and excluded *Zuvanda* from *Malcolmia* and *Maresia*. Nevertheless, they failed to resolve the tribal assignment of *Zuvanda* and erroneously suggested performing some analyses to check the affinity of this genus with Isatideae. Later on, German and Al-Shehbaz generated a new tribe named Conringieae and placed *Conringia* and *Zuvanda* in it [18]. However, this was not the end of this story and German unjustified the name *Zuvanda *[45]. He discovered that the name *Plagioloba* Rchb. was initially adapted for this genus and then subsumed the species of this genus into two (*P. crenulata* (DC.) D.A. German and *P. meyeri *(Boiss.) D.A. German). He also defined two varieties for *P. crenulata* (*P. crenulata* var. *crenulata* and *P*. *crenulata* var. *exacoides* (DC.) D.A. German).

The current study deals with the taxonomic status of *Arabis ottonis-schulzii* along with *Conringia *and* Plagioloba *species inferred from nuclear ribosomal DNA sequence dataset (ITS_1_ and ITS_2_), seed coat microsculpturing, and the nature of septum cells together with morphological data. 

## MATERIALS AND METHODS

**Study group:** To infer the phylogenetic position of *Arabis ottonis-schulzii* within Brassicaceae, we included a broad sampling of Brassicaceae representing nearly all tribes of the family in the old world [see 38]. The representative of Arabideae and Conringieae were also selected due to the placement of *Arabis *and *Conringia* within these tribes, respectively. *Aethionema *was also used as the outgroup. Indeed, the first author (ARKH) critically examined the type specimen, considerable herbarium samples of *Arabis ottonis-schulzii* in valid virtual herbaria (e.g., RBGE and W), Iranian Herbaria like Herbarium of Shiraz University (HSHU), the Research Institute of Forests and Rangelands (TARI), as well as fresh plants in field studies.

It has been tried to obtain nrDNA sequence of studied specimens from herbarium or fresh plant materials in Shiraz University lab (*Aethionema erinaceum *Thell., *A.* *carneum *B. Fedtsch,* Anastatica hierocontica *L., *Conringia orientalis *(L.) Dumort, *Conringia clavata *(DC.) Link, *Arabis* *ottonis-schulzii*, *Dielsiocharis kotschyi *O.E. Schulz, *Alyssopsis mollis *(Jacq) O.E. Schulz, *Alyssum desertorum *Stapf*.*, *Farsetia heliophila *Bunge ex Coss*.*, *Spirorhynchus sabulosus *Kar. & Kir., *Goldbachia laevigata *(M. Bieb.) DC., *Graellsia saxifragifolia *(DC.) Boiss, *Didymophysa aucheri *Boiss., *Brassica deflexa *Boiss*.*, *Sisymbrium leucocladum *(Boiss.) D.A. German & Al-Shehbaz, *Isatis* *brevipes* (Bunge) Jafari and *Iljinskaea* *planisiliqua*), if not they are obtained from online repositories such as NCBI (https://www.ncbi.nlm.nih.gov/). Moreover, we have been used different specimens of *Conringia persica* (three) and *Arabis ottonis-schulzii* (five) to clarify their taxonomic position accurately. The validity of the sequences was checked carefully using The Basic Local Alignment Search Tool (BLAST) in NCBI. The studied species have already been listed in Online Resource 1. Moreover, tribal assignments were followed from Brassibase: https://brassibase.cos.uni-heidelberg.de/?action= phylo/ [46]. 

**DNA extraction, amplification, and sequencing**. Total DNA was isolated from fresh and dry material using the CTAB protocol [47]. Double-stranded DNA of the complete ITS region, including the 5.8 S rDNA gene, was amplified by 35 cycles of PCR using ITS primers described in [48]. The PCR profile was: 5 min at 94˚C, and 35 cycles of amplification (1 min 94˚C, 45 s 38˚C, 45 s 72˚C), final elongation step for 10 min at 72˚C, and storage at 4˚C. PCR products were purified using the Boehringer PCR product purification kit (Roche Molecular Biochemicals). Sequencing reactions were run on an ABI 377XL automated sequencer in MWG DNA Sequencing services (Ebersberg, Germany). 

**Figure 1:**
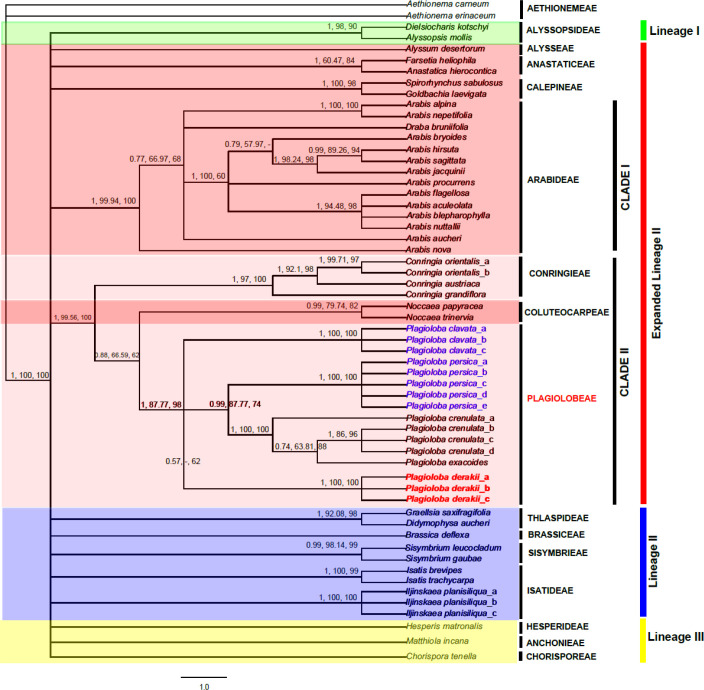
The results of the phylogenetic analysis of ITS sequence is displayed herein based on a Bayesian approach. Numbers above 50% majority rule consensus tree are refer to Bayesian posterior probability (PP), maximum parsimony (MP) and maximum likelihood (ML) bootstrap values, respectively (right to left). Each single specimen used in the current study nested within a tribe, which are clearly written in front of each taxon. Lineages (I-III) showed by different colored boxes. New combinations highlighted with a blue color, while new introduced tribe and species are marked with red

**Phylogenetic analyses:** DNA sequences were checked and aligned manually using MEGA software ver. 10.2.6 [49]. The best substitution evolutionary model was obtained from AIC (Akaike Information Criterion) [50] in jModelTest2 on XSEDE v. 2.1.6 in the CIPRES Science Gateway v.3.3 [51]. The best model was selected as follows: SYM+I+G. The aligned ITS sequences were subjected to Bayesian (BI), maximum parsimony (MP) and maximum likelihood (ML). BI and ML analyses were performed in MrBayes Restart on XSEDE (3.2.x) and RaxML-HPC BlackBox (8.2.12) in CIPRES [51-52], respectively. MP analysis was also applied in PAUP*4.0b10 [53]. The setting of all analyses was followed Eslami-Farouji et al. [54]. Finally, trees were checked in FigTree v.1.4.3. BI, MP and ML results were summarized by 50% majority rule consensus tree and obtained posterior probability (PP), MP and ML values (‘‘clade credibilities’’) are indicated at the branches, respectively (Fig. 1).

**Morphological studies:** Due to the presence of homoplastic morphological characters in Brassicaceae, only limited features are recommended being used at tribal and genera levels [e.g., 55]. However, not only morphological studies but also micromorphological investigations lead to a better understanding of studied specimens. Thus, we provided morphological study as well as scanning electron micrographs from studied genera (*Arabis*, *Conringia* and *Plagioloba*). 

In the case of SEM observations, various seeds were examined and the best mature ones were chosen for further analyses. To observe the seed-coat microcharacters, they were directly mounted on metal stubs using plastic conductive carbon cement and sputter-coated with gold. Observations were performed by a Zeiss, DSM 960 microscope at an accelerating voltage of 20 kV in Tehran University. Micromorphological terminology followed some literatures [e.g., 56-57]. 

Furthermore, septum images were also prepared to show the nature of septum cells. Parts of the septum were taken from the middle part of the fruit. The best specimens were examined, without staining, by using a light microscope (Olympus microscope model CH40) and were photographed with a Canon camera EOS 5DS R from non-permanent slides.

## RESULTS

Statistical summary of nrDNA ITS within studied specimens has already been listed in Table 1. The result of the phylogenetic analysis of ITS sequence variation using the Bayesian approach is shown in Fig.1. 

The Bayesian tree topology is almost identical with ML and MP 50% majority rule consensus trees (Fig. 1, two latters not shown here). Generally, the topology of our tree is completely congruent with what Kiefer et al. discussed in Brassibase: https://brassibase.cos.uni-heidelberg.de/?action=phylo/ as a standard phylogenetic graph in Brassicaceae [46]. 

**Table 1:** Statistical characteristcs of nuclear ribosomal ITS in studied taxa

**Statistical characters**	**ITS**
Number of sequences	57
Alignment length	693
Tree length	1283
Number of parsimony informative characters	280
Variable sites	134
Retention index (RI)	0.72
Consistensy index (CI)	0.49
Homoplasy index (HI)	0.50

The main purpose of this study is chiefly focused on expanded lineage II (see Fig. 1). As a result, the remaining lineages (I, II & III) do not discuss further. Expanded lineage II (indicated by red color, Fig. 1) clearly divided into two well-defined clades with high clade credibilities as follows: **CLADE I** (PP/ MP & ML: 1, 99.94 & 100) including members of tribe Arabideae, while **CLADE II** (PP/ MP & ML: 1, 99.56 & 100) comprising tribes Conringieae and Coluteocarpeae) (Fig. 1). Tribe Conringieae uncovers a paraphyletic group due to the position of tribe Coluteocarpeae (*Noccaea papyraceae* (Boiss.) Khosravi, Mumm. & Mohsenz. and *Noccaea trinervia* (DC.) Steud.). This study suggests that *Conringia* species should be split into different genera and species. In the case of new combinations, we are following German study regarding the name *Plagioba* as a legal name for *Zuvanda*, and thus justified this name for the second clade (Fig. 1) [45]. Likewise, the phylogenetic analyses positioned an overlooked *Arabis ottonis-schulzii* plus *C. persica* as two indistinct entities (CLADEII, Fig. 1) within tribe Conringieae. 

Moreover, the true position of two *Conringia* species (*C. clavata *and* C. persica*) unraveled. In this case, the number of species within a monogeneric tribe Conringieae reduced into three (*Conringia austriaca *(Jacq.) Sweet, *C. orientalis* and *C. grandiflora *Boiss. & Heldr.). 

**Figure 2:**
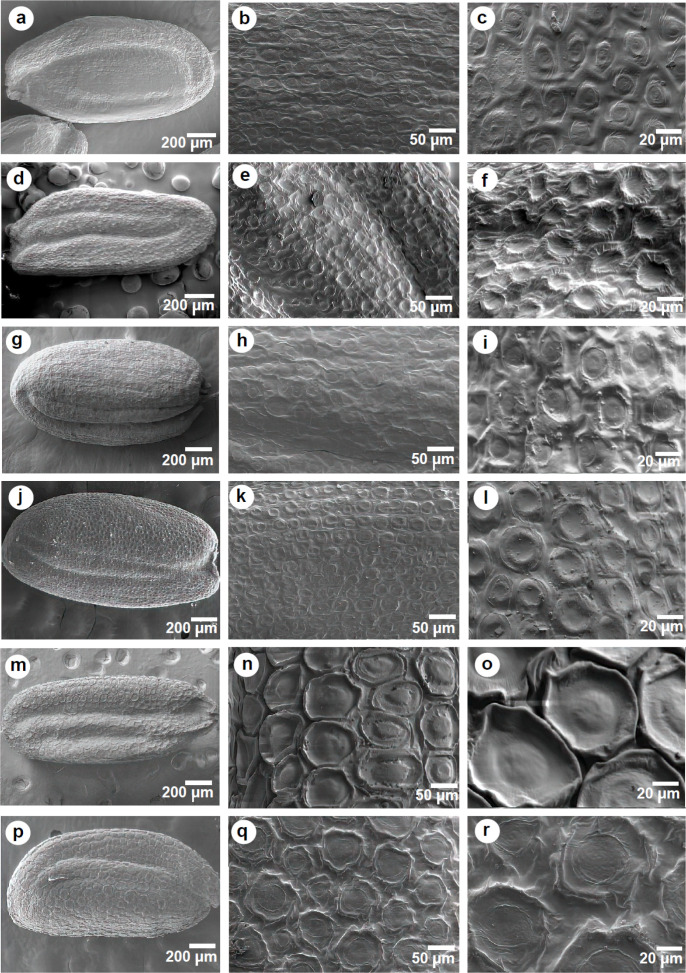
Seed surface micro-sculpturing of Iranian *Conringia*, *Plagioloba* and *Iljinskaea* studied taxa. **a-c:** *P. persica*; **d-f:** *P. crenulata*; **g-i:** *P. derakii*; **j-l:** *P. clavata*;** m-o:** *C. orientalis*; **p-r:** *I.* *planisiliqua*

Seed micromorphological graphs (Fig. 2) provided striking results within studied lineages*.* Two types of seed surface ornamentations were determined at low magnification (×50): Reticulate in *Arabis ottonis-schulzii *(*C. persica* variant I), *Conringia clavata,* *C. persica *(*C. persica* variant II) and* Plagioloba crenulata, *and ocellate in *C. orientalis *and *Iljinskaea planisiliqua. *However, at higher magnifications (×200, ×500), these could be further divided into three patterns: 1) reticulate with ocellate structure in *A. ottonis-schultzii *(*C. persica* variant I), *C. clavata, C. persica *(*C. persica* variant II) and* P. crenulata*; 2) domate with central structure in *C. orientalis*; 3) domate without central structure in *I. planisiliqua*. The epidermal cells are larger in *C. orientalis *and *I. planisiliqua *than other species. In *A. ottonis-schultzi *(*C. persica* variant I), *C. clavata, C. persica *(*C. persica* variant II) and* P. crenulata *the epidermis cells are smaller, forming a pusticulate-foveate pattern with marked slime body rings and a central crater. Although *C. orientalis *and *I. planisiliqua* show the ocellate type of seed coat ornamentation, they differ in the nature of the cells. In *C. orientalis *seed coat cells are characterized by domate with central structure, while *I*.* planisiliqua* have flat periclinal cell walls. 

The anticlinal cell wall boundaries show variation between genera and species. Except in *A. ottonis-schultzi *(*C. persica* variant I) with the raised-channeled anticlinal cell wall, other taxa have channeled anticlinal wall structure with different depth and width. (Fig. 2). Anticlinal cell boundaries of *C. orientalis* were sunken/deeply channeled, and because of that, they were clearly different from all other examined taxa. *Iljinskaea planisiliqua* was characterized by sunken/flat anticlinal cells. The outer periclinal cell walls are concave or slightly concave in *A. ottonis-schultzi *(*C. persica* variant I), *C. clavata, C. persica *(*C. persica* variant II) and* P. crenulata, *while it is clearly convex in *C. orientalis *and flat in *I. planisiliqua.*

**Figure 3:**
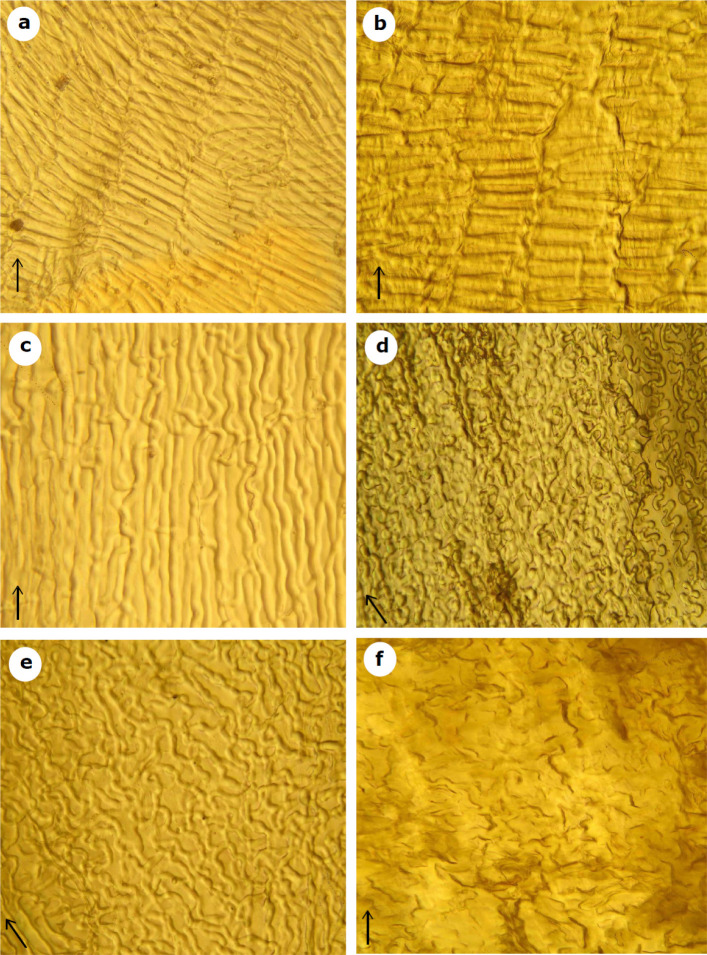
Septum surface of Iranian *Conringia*, *Plagioloba* and *Iljinskaea *studied taxa. a: *I.* *planisiliqua*; b: *C. orientalis*; c: *P. clavata*; d: *P. derakii*; e: *P. persica*; f: *P. crenulata*. The fruit axis marked with black flash. Scale bar for all images is ×400. Images are prepared and taken by the first author (ARKH)

Phenotypic variation within septa of studied taxa, and their close relatives were studied for the first time and clearly identified four types (Fig. 3): In the septum two types of surface cellular arrangement were determined. In *C. orientalis *and *I. planisiliqua, *the epidermal cells are perpendicular to the long axis of the fruit, while in *A. ottonis-schulzii *(*C. persica* variant I), *C. clavata, C. persica *(*C. persica* variant II) and* P. crenulata* are parallel. Epidermal cell shapes in the septum can be categorized into four groups: The first group is mainly comprising very long oblong cells with blunt or tapering end walls and striate anticlinal thin wall (*I. planisiliqua*, Fig. 3a), while the second group has long oblong cells with blunt end walls and striate anticlinal thick walls (*C. orientalis *(Fig. 3b); The third septum type is belonging to *P. clavata* with very long oblong cells and blunt end walls and sinuous anticlinal thick wall (Fig. 3c). Finally, the fourth group finds out in *A. ottonis-schulzii *(*C. persica* type I), *C. persica *(*C. persica* type II) and* P. crenulata *with very long oblong cells, blunt end walls and undulated anticlinal thick wall. 

## DISCUSSION

**Figure 4:**
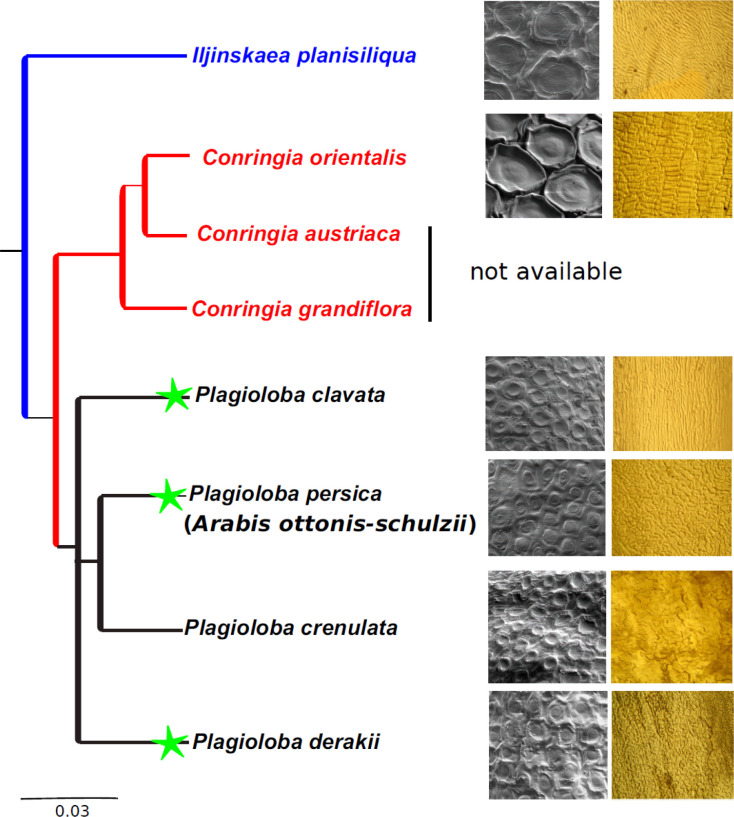
New tribe Plagiolobeae together with new combinations (*P. persica*; *P. clavata*). The species of each tribe indicated by a distinct color (**black**: Plagiolobeae; **blue**: Isatideae and **red**: Conringieae). Moreover, new species and combinations are demonstrating by green stars

**Figure 5:**
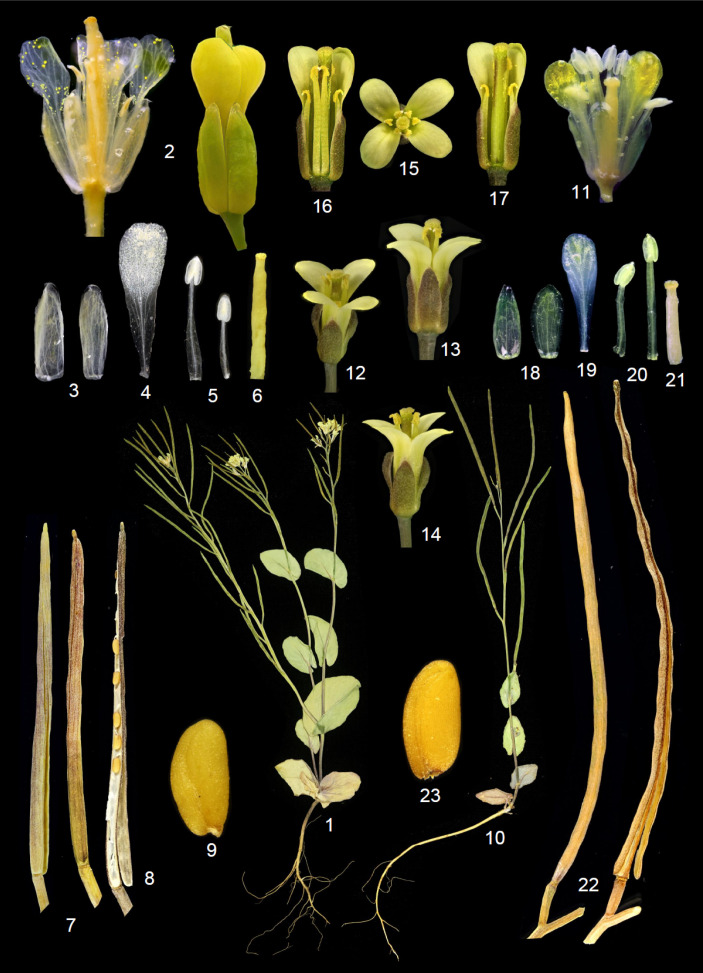
Morphological characteristics of *Plagioloba* *derakii* and *P. persica* species in Iran. **1-9:** *P. derakii* *sp. nov.* (**1:** Life form, **2:** flower, **3:** sepal, **4:** petal, **5:** anther, **6:** Gynoecium, **7:** fruit, **8:** a fruit with one valve removed, **9:** seed); **10-23:** *P. persica *(**10:** life form, **11-14:** flower, **15:** flower top view; **16-17:** a flower with one petal and two stamens removed, **18:** sepal, **19: **petal, **20:** anther, **21:** Gynoecium, **22:** fruit, **23:** seed). Images are photographed by the first author (ARKH)

In the current study, we use evidence from nuclear ITS sequences, micromorphological data together with morphology in order to clarify the generic status and the phylogenetic relationships of *A. ottonis-schulzii*, a morphologically enigmatic species in the genus *Arabis *plus Conringieae. Our molecular study clearly shows that *A. ottonis-schulzii *is not nested within Arabideae, and clarified its phylogenetic position within paraphyletic Conringieae*.* Furthermore, the studied taxon demonstrated its close phylogenetic relationship with *Plagioloba*. As recently discussed, the first author (ARKH) morphologically recognized two variants of *C. persica. *The first is totally similar to the type of *C. persica* collected by Kotschy in 1842 (339!), but the second variant of *C. persica* is considered to be new due to molecular data (Fig. 1) as well as morphological characters (Figs. 2-5). We have sampled and later sequenced both variants from Kuh-e Barfi, as Kotschy collected before.

The number of *Arabis *species were decreased through time by phylogenetic studies [e.g., 2, 3, 5 and references therein], into various genera such as *Arabidopsis *Heynhold*, Boechera* Á. Lӧve & D. Lӧve*, Catolobus *(C.A. Mey.) Al-Shehbaz*, Fourraea *Greuter & Burdet*, Pseudoturritis *Al-Shehbaz*, Rhammatophyllum *O.E. Schulz*, Streptanthus *Nuttall within about five tribes [e.g., 11]. According to Al-Shehbaz, latiseptate fruits, accumbent cotyledons and furcate trichomes are the most diagnostic characters for *Arabis *delimitation [58]. A critical comparison of the *Arabis *s. str. species and *A. ottonis-schulzii *(*C. persica *variant I) reveals that there are a number of morphological characters that readily distinguish the *Arabis* species from *A. ottonis-schulzii *(*C. persica *variant I). Based on the current study, *A. ottonis schulzii* (*C. persica *variant I) is a glaucous glabrous plant that almost characterized by falcate long delicate fruits, small dark green or greenish-violet perfoliate leaves, yellow or pale yellow petals and incumbent cotyledons. Generally, the original description of *A. ottonis-schulzii* (*C. persica *variant I) include several inaccuracies; for instance, the petals described as white in color in Flora Iranica [59], while the observations confirmed that the petal of the mentioned taxon is exclusively distinguished by yellow or pale yellow color. Indeed, white color is the diagnostic character for *Arabis* specimens. Moreover, *A. ottonis-schulzii* (*C. persica *variant I) differs from the remaining *Arabis* species by presence of slightly saccate sepals and falcate fruits. The interspecific taxonomic delimitation of* A. ottonis-schulzii* has some confusions too; e.g., *A. ottonis-schulzii* (*C. persica *variant I) and *C. persica* (*C. persica *variant II) are glabrous and glaucous with almost fleshy perfoliate stem leaves [60]. As a result, except for having curved fruit and small darker green leaves, *A.* *ottonis-schulzii* (*C. persica *variant I) is almost similar in all aspects of leaves and flowers to *Conringia persica *(variant II) (Fig.1). Two mentioned variants were almost identified under the name *C. persica* in some Iranian (e.g., FUMH, TARI, IRAN) and foreign herbaria (e.g., RBGE and W) as both *Conringia persica* variants with different characters described as *C. persica* in Flora of Iran [17]. 

Geographically, the two mentioned species are distinct. *Arabis ottonis-schulzii* (*C. persica *variant I) is mainly distributed in the south, central Iranian plateau and Afghanistan, while *C. persica* (*C. persica *variant II) is growth in west of Elburz, western slope of Zagros, N of Turkey, the Caucasus along with north of Iraq. With respect to their geographical distribution, their ecological environments are critically differing from each other as *A. ottonis-schulzii* (*C. persica *variant I) resistant more to dry environmental conditions (xerophytic) than *C. persica* (mesophytic) (*C. persica *variant II). 

As mentioned before, *Conringia* was nested within tribe Brassiceae by numerous authorities (see introduction). Based on what Al-Shehbaz mentioned in his paper [29], *Conringia persica* comprises the shortest flowers among *Conringia* species with non-saccate sepals, and this study is completely in agreement with the former but not with the latter idea. To our knowledge, he described *C. persica* (variant I, in our study), while we found slightly saccate sepals in both variants of *C. persica*. Regarding *C. persica* (variant I), Anderson and Warwick exclude *Conringia* from their study and support the monophyly of Brassiceae [61]. However, Warwick and Sauder strongly confirmed the close relationship of the genus *Conringia* with Brassiceae [30]. Bailey et al. generated a well-defined study regarding Brassiceae and disclose non-monophyly of *Conringia* within this tribe [32]. They also showed both *C. clavata* and *Noccaea* Moench as closely related species, and proved the strong affinity of *Conringia* (e.g., *C. clavata* and *C. orientalis*) to the tribe Coluteocarpeae, as showed in previous papers [1, 35, 62, 63, 64, 65], and this study. Beilstein et al. established a phylogenetic study based on *ndhF*, *PHYA*, as well as combined dataset with high and low levels of Bayesian and bootstrap supports, respectively [34]. They claimed that Coluteocarpeae is monophyletic, and supported the affinity of it with *C. persica* and *C. clavata*. Their well-established molecular analyses were also suggested the inclusion of *C. persica* and *C. clavata* to the tribe Coluteocarpeae. However, the present study confirmed the evolutionary affinity of tribes Noccaeeae and paraphyletic Conringieae (Expanded lineage II). Generally, Beilstein et al. also suggested that it is possible to transfer *C. persica*, *C. clavata* and maybe other members of *Conringia* to Noccaeeae [34], which is completely in contrast with our idea. We assume that the cause of this misleading idea is due to the absence of *P. crenulata* varieties and *A. ottonis-schulzii* (*C. persica *variant I) specimens in their study. The present study undoubtedly distinguishes *Plagioloba* as a monophyletic taxon (see Fig. 1). 

German et al. and our study clarified the close relationship between *Plagioloba* and some species of *Conringia *[27]. It also stated that Coluteocarpeae has a distinct taxonomic position from *Conringia* in terms of morphological features such as silique and stigma shape and frequency of seeds in each single fruit [27]. Koch and Marhold neglected a few insufficiently known *Arabis* specimens (e.g., *Arabis ottonis-schulzii*) in their study and assumed that they resolved delimitation of *Arabis* by introducing three different genera [66]. According to Özüdoğru and his co-workers, in contrast with ITS results, *trnL-F* failed to support the monophyly of Coluteocarpeae and *C. orientalis* (Conringieae) [67]. They claimed that the monophyly of Coluteocarpeae remains unresolved, while molecular data proved the whole lineage (Coluteocarpeae and *Conringia*) as monophyletic. Nikolov et al. highlighted the close relationship of *Noccaea vesicaria *(L.) Al-Shehbaz, *Arabis ottonis-schulzii* and *Conringia orientalis*, but their data were not sufficient to explore the tribal assignment and taxonomic circumscription of *Arabis ottonis-schulzii *[15], as we did.

Regarding *Plagioloba*, Warwick et al. were molecularly studied *Malcolmia* complex and noticed the distinct taxonomic position of *Plagioloba *[44]. Nevertheless, they were unable to assign *Plagioloba* to a specific tribe. Later studies highlighted the close affinity of *Plagioloba* and *Conringia* and strongly supported the placement of *Plagioloba* within Conringieae [27, 68]. Al-Shehbaz et al. subsequently referred to distinct morphological differences of *Malcolmia* and *Plagioloba *(presence of auriculate to sagittate stem leaves and absence of furcate trichomes are in *Plagioloba*) [69].

**Micromorphological studies**: It is believed that seed microsculpturing is a significant character to identify species [e.g., 70-72]. Seed characters have been used in tribal and subtribal delimitations [e.g., 13, 22, 33], evolutionary classification [73], as well as traditional sectional circumscription [e.g., 72]. However, attention to the seed structure of some species may misleadingly guide authors to put two unrelated specimens in the same group [e.g., 74]. 

The relevance of seed coat characters was not supported in the delimitation of genera in the study of Moazzeni et al. [75], while Kasem et al. revealed the significance of seed characters in generic and intrageneric levels such as shape, color, seed coat microsculpturing, anticlinal and periclinal walls [56]. Based on what we studied in the present survey, characters such as color, size and shape are not phylogenetically useful in evaluating taxonomic relationships. Stork and Kaya et al. were taxonomically tried to separate *Malcolmia*, *Plagioloba* and *Strigosella* Boiss. based on seed micromorphology [76-77]. Their project together with the present study declared the use of seed surface patterns along with anticlinal and periclinal cell walls in the separation of studied taxa (Figs 2-4). Özüdoğru et al. also stated that LM and SEM investigation of seed characters reveals the taxonomic importance of seeds [78]. They successfully concluded the correspondence of seed shape characters with phylogenetic results. The importance of seed characters in tribal assignments was revealed by Gabr [79], and our project highlighted the importance of seed coats in diagnosing the studied lineages (e.g., Isatideae, Conringieae).

Except for some investigations [e.g., 1, 20, 21, 69, 80, 81, 82, 83], septum did not much study in taxonomic delimitations of Brassicaceae. Not only earlier surveys neglected such kind of characters [84], but also later researchers criticized some morphological characters like fruit septum due to considerable variations [69, 85]. Dvořák found out the heterogeneity in septum cells, and remarkably pay attention to fruit septum in *Malcolmia* complex [80], but Koch studied some separating morphological characters (e.g., fruit septum) within *Ionopsidium *Rchb. [81]. Al-Shehbaz et al. were only described the septum transparency (hyaline or opaque) and their thickness [69]. Then, Ali et al. conducted a morphological study regarding septum cells in Brassicaceae (e.g., *Friedrichkarlmeyeria umbellata *(F.K. Mey.) Tahir Ali & Thines, *Ihsanalshehbazia granatensis *(Boiss. & Reut.) Tahir Ali & Thines) [82]. As we proved the significance of fruit septa in studied specimens in Brassicaceae, they also claimed that septum cells have a spindle shape in *Friedrichkarlmeyeria umbellata*, while *Ihsanalshehbazia granatensis* has different septum nature. However, none of above mentioned papers did not prepare highly transparent septum cells and only superficially studied fruit septa. In contrast with modern molecular workers who almost trust the molecular dataset, we broke from tradition when we tried our best to examine septum cells (Fig. 3). 

As the first author collected several *C. persica* (variants I and II) in his field trips during ten years, he found out that *A. ottonis-schulzii* (*C. persica* variant I) and *C. persica* (variant II) are the same species. Our detailed morphological studies together with molecular data, confirmed this statement as previously did by Assadi et al. [17]. We might also have documented this idea by evaluating Kotschy’s specimens (*C. persica* variant I) with *C. persica* (variant II), which are collected from Iran, Turkey and Iraq. They are morphologically differing from each other (e.g., leaf, flower and fruit morphology). The type specimens which were collected by Kotschy was *A. ottonis-schulzii*, which already synonymized under *C. persica* (variant I). Thus, the current study supports the removal of *Arabis ottonis-schulzii* (*C. persica *variant I) as a member of Arabideae and justified *Plagioloba persica* (Boiss.) A.R. Khosravi & A. Eslami-Farouji, *comb. nov. *as a new combination. Moreover, we propose the new combination of *Plagioloba clavata* ((DC.) Link) A.R. Khosravi & A. Eslami-Farouji *comb. nov*,*.* A new species, *Plagioloba derakii* A.R. Khosravi & A. Eslami-Farouji *sp. nov.* (*C. persica* variant II), is also described due to molecular, morphological and micromorphological results (Figs 1-5). Eventually, the new tribe Plagiolobeae A.R. Khosravi & A. Eslami-Farouji *trib. nov.* suggested as one additional tribe in family Brassicaceae comprising five species (*P. clavata*, *P. derakii*, *P. crenulata*, *P. persica *and* P. meyeri*), and seed and septum micromorphological data critically supports our idea (see Figs. 1-4).

**Taxonomic considerations: **Tribe Plagiolobeae A.R. Khosravi & A. Eslami-Farouji *trib. nov.* Type: *Plagioloba* Rchb., Deut. Bot. Herb. -Buch: 182. 1841. *Annual herbs; glabrous or with simple minute trichomes; cauline leaves perfoliate, entire or dentate, basal leaves petiolate; petals yellow or pale yellow, lilac, or white; ovules numerous; fruits siliques, glabrous or with tuberculate hairs; stigmas decurrent or capitate; septum cells parallel with fruit axis; seeds uniseriate; radicle incumbent or oblique incumbent, seed numerous; seed coat with reticulate sculpture.*

**Notes**:—The main difference between tribes Plagiolobeae and Conringieae is defined by the smaller size (vs. larger size) of periclinal cell walls and the parallel (vs. perpendicular) epidermal cells direction to the long axis of the fruit septum in Plagiolobeae. Moreover, the size of periclinal cells in seed coat is smaller (vs. larger) in tribe Plagiolobeae (see Figs. 2-4).

**Figure 6:**
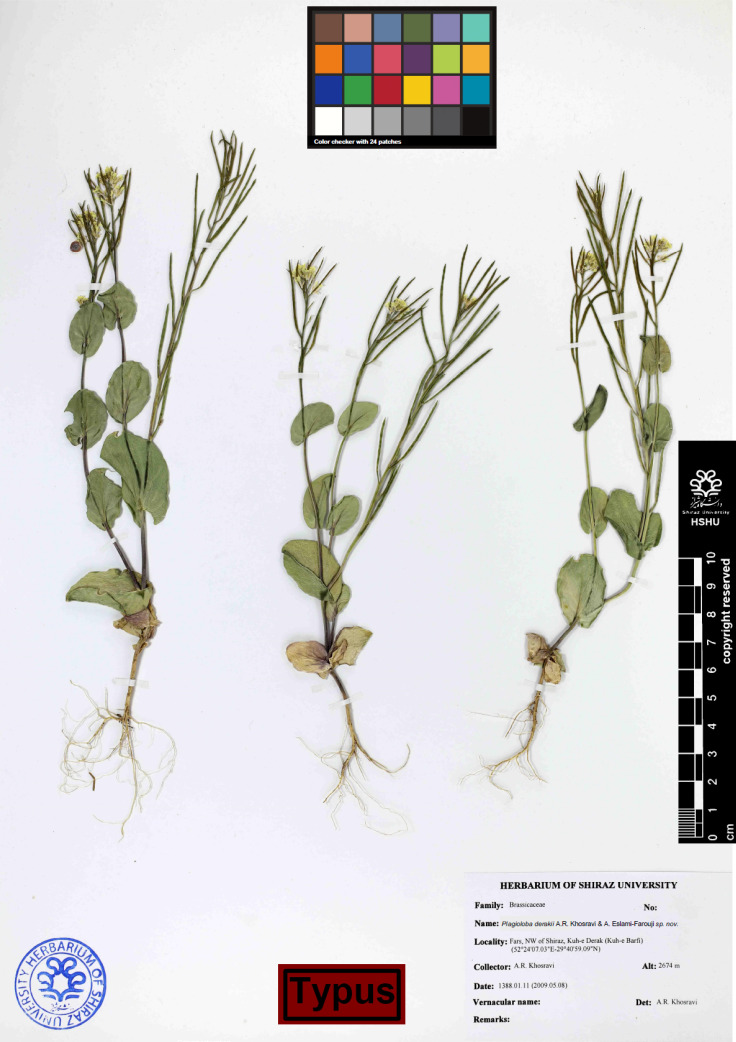
Herbarium specimen including *Plagioloba derakii* A.R. Khosravi & A. Eslami-Farouji *sp. nov. *Holotypus

The tribe includes *Plagioloba* (5 spp.) as follows:

***Plagioloba derakii*** A.R. Khosravi & A. Eslami-Farouji ***sp. nov.*** —Figs. 2, 3, 5 & 6.—**HOLOTYPE**:—Persia, Fars Province, NW of Shiraz, Kuh-e Barfi (Kuh-e Derak), 2674 m, (29°40'59.9"E-52°24'07.3"N), 2009.03.31, *A.R. khosravi 42048* (HSHU). 

**Etymology**:—The epithet ‘derakii’ refers to the type species location, Derak Mountain.

**Description**:—Plant annual. Stems ascending to erect, 5-20 cm long, simple or branched from base, often violet stemmed. Basal leaves entire, cuneate, cauline leaves deeply cordate, amplexicaul, pedicels 2-4 mm. Sepals oblong, ascending, the inner pair slightly saccate, 3–4.5 × 0.5–1 mm; petals yellow, 3.5-4 mm long, obcuneate. Fruiting pedicels 0.4-1.2 cm., ascending, thickened. Siliqua erect-spreading or loosely appressed to stem, 20-50 × 1-1·5 mm, linear, subterete, with a very short punctiform stigma; nerves several, indistinct. Seeds pale brown, oblong, 1.2 mm long, 0.5 mm broad with reticulate sculpture. (Figs. 2-3).

**Diagnosis**:—The new species is closely related to *P. persica* morphologically, but differs from it by having larger perfoliate light green stem leaves, (vs. smaller perfoliate greenish violet stem leaves), larger flowers up to 5 mm (vs. smaller flowers up to 4 mm), fruits mostly erect up to 5 cm (vs. fruit mostly curved up to 7 cm).

**Phenology**:—April-June (flowering period), June-July (fruiting time).

**Distribution area**:—W Iran, E Turkey and N Iraq & Caucasus.

**Additional specimens examined**:—Iran: Kurdistan, 16 km N of Husainabad between Sanandaj and Saqez, exposed hill of upland plateau, 2160 m, 21 May 1996, *J.C. Archibald 2114* (RBGE!). –Iran: Azerbaijan, frontier of Turkey beyond Qotur, 2000-2100 m, 10 June 1971, *Coll. Jennifer Lamond 3946* (RBGE!) – Turkey: B9 Agri, 2 km SW of Hamur (Murat valley), Colonising earthy banks in steppe. 1670 m, 02 June 1966, *Davis 44034* (RBGE!). –Turkey: Prov. Kars, Fallow field on plain, 1800 m, 15 Jun 1957 (RBGE!). Iran: Fars, 58 km W of Shiraz, 3600 m, 16 May 1964, *Martin L. Grant 15542* (HSHU!); Iran: Azerbayjan, road of Oshnaviyeh to Urmia, 08 Jun 2009, *A.R. Khosravi & Assadollahi* (HSHU!) 

**Proposed conservation status:**—According to IUCN Red List category [86], an invulnerable status is proposed for *P. derakii.*

***Plagioloba persica*** (Boiss.) A.R. Khosravi & A. Eslami-Farouji ***comb. nov*.** Basionym: *Conringia persica* Boiss., Diagn. Pl. Or. Nov. Ser. 1, 6: 12 (1845). Type: —IRAN. Prov. Fars: Shiraz, Kuh-e Barfi, KY. 339! 1842.05.04, T. Kotschy, 339 (holotype K!). Syn.: *Arabis ottonis-schulzii *Bornm. & Gauba, Feddes Repert. 39: 80. Tab. 198a (1935). —Figs. 2, 3 & 5.

**Distribution**: —South, central Iranian plateau and Afghanistan.

***Plagioloba clavata*** (Boiss.) A.R. Khosravi & A. Eslami-Farouji ***comb. nov.***—Figs. 2 & 3. Basionym: *Conringia perfoliata* (C.A.Mey.) N.Busch, Komarov, Fl. URSS 8: 497 (1939). Type: —IRAN. Prov. Gilan, Talish prope Swant, C.A. Mey., (holotype LE!). Syn.: *Sisymbrium perfoliatum *C.A. Mey., Verz. Pfl. Cauc. 188 (1831); *Conringia clavata* Boiss., in Ann. Sci. Nat. 17: 84 (1842). 

**Distribution**:—Syria, Lebanon, Turkey, Caucasus, Iran, Afghanistan, C. Asia (Turkmenia).

***Plagioloba crenulata*** (DC.) D.A. German 

***Plagioloba meyeri*** (Boiss.) D.A. German 

## Conflict of Interest

Authors have a financial relationship with the organization that sponsored the research. 
